# Uptake of Radionuclides ^60^Co, ^137^Cs, and ^90^Sr with α-Fe_2_O_3_ and Fe_3_O_4_ Particles from Aqueous Environment

**DOI:** 10.3390/ma14112899

**Published:** 2021-05-28

**Authors:** Natalya V. Efimova, Alla P. Krasnopyorova, Galina D. Yuhno, Dmitry S. Sofronov, Mirosław Rucki

**Affiliations:** 1 Department of Radiochemistry and Radioecology, V.N. Karazin Kharkiv National University, 4 Svobody Sq., 61022 Kharkiv, Ukraine; n.v.efimova@karazin.ua (N.V.E.); alla.p.krasnopyorova@karazin.ua (A.P.K.); g.yukhno@karazin.ua (G.D.Y.); 2 State Scientific Institution «Institute for Single Cristal», National Academy of Sciences, 60 Nauky Avenue, 61001 Kharkiv, Ukraine; sofronov@isc.kharkov.com; 3 Faculty of Mechanical Engineering, Kazimierz Pulaski University of Technology and Humanities in Radom, ul. Stasieckiego 54, 26-600 Radom, Poland

**Keywords:** α-Fe_2_O_3_, Fe_3_O_4_, radionuclides, sorption, strontium, cesium, cobalt, drinking water

## Abstract

In the paper, investigation results of the uptake efficiency of radionuclides ^60^Co, ^90^Sr, and ^137^Cs dissolved in water onto iron oxides α-Fe_2_O_3_ and Fe_3_O_4_ are presented. It was found that sorption efficiency increased for higher pH values. Independent of the oxide nature, the uptake characteristics are the best toward ^60^Co and the worst toward ^137^Cs, forming the row as follows: ^60^Co > ^90^Sr > ^137^Cs. The highest sorption ability at pH 9 was found for magnetite Fe_3_O_4_, which was 93%, 73%, and 26% toward ^60^Co, ^90^Sr, and ^137^Cs, respectively, while the respective percentages for hematite α-Fe_2_O_3_ were 85%, 41%, and 18%. It was assumed that the main sorption mechanism was ion exchange. That may explain some decrease of the sorption efficiency in drinking water due to the interfering presence of magnesium and calcium cations. The obtained results indicated the feasibility of the tested sorbents and their merits, especially in terms of relatively high uptake coefficients, low costs, availability, and lack of toxicity.

## 1. Introduction

The necessity of the ecological control of the aquatic environment in terms of radionuclides pollution level is closely connected with the activity of nuclear energetic complexes and especially their accidental failures. Special attention is paid to the consequences of catastrophic accidents at the Chernobyl nuclear plant in 1986, and at the Fukushima Dai-ichi Nuclear Power Plant in 2011, e.g., in papers [1,2]. According to the WHO Guidelines [3], the maximal acceptable total volumetric activity of *β*-radiators, i.e., Σ*β* activity, should not exceed 1.0 Bq/dm^3^. The main portion of the total specific activity in the polluted waters belongs to the long-term radionuclides ^137^Cs and ^90^Sr. In the aquatic environments, these ones are present in the form of exchangeable radionuclides (cationic and water-soluble complex), as described by several authors [4,5]. Izrael et al. [6] emphasized that the radionuclides promoted their intensive migration in biosphere and threat to the human health. Gupta et al. [7] found that the radioactive strontium enters the human food chain by the consumption of plants, while according to Rauwel and Rauwel [8], after the consumption of contaminated game and fish, ^137^Cs is introduced to the human body and emits radiations directly affecting the cell nucleus. Recent studies [9] demonstrated the transport of ^137^Cs from nuclear reprocessing plants Sellafield and La Hague, as well as from Chernobyl accident to the Baltic Sea. Similarly, Kurikami et al. [10] suggested its transport from the forests to freshwater fish living in mountain streams in Fukushima. Cobalt belongs to the group of heavy metals, i.e., trace elements with elemental density above 4 ± 1 g/cm^3^. At dosages higher than the prescribed WHO limits, these substances are considered toxic to soil and aqueous systems, as it was emphasized in [11]. Demir et al. [12] reported that observations of people accidentally exposed to a ^60^Co source confirmed a significant unfavorable change induced by radiation in the oxidant/antioxidant status in their organisms. Therefore, many researchers emphasize the importance of cobalt removal from waters and soil [13,14].

Thus, it is urgent and important to improve the existing methods and to develop new ones in order to perform effective deactivation of radionuclide-polluted natural reservoirs and wastewaters. The simplest, most easily available, and effective methods are those based on the sorption phenomenon described in [15], since large volumes of water can be treated without affecting its chemical composition and adding new contaminations. The present study is focused on the uptake of ^60^Co, ^90^Sr, and ^137^Cs radionuclides. A wide range of materials has been proposed, so that a particular one may be chosen dependent on the removal effectiveness, chemical stability, expenses, regeneration ability, etc. described in details e.g., in [16,17]. Among numerous recent reports, it is worth mentioning the study of polyaniline/zirconium aluminate composite (PANI/ZAl) as a novel sorbent synthesized by the sol–gel hydrolysis method. According to Metwally et al. [18], its capacity was 140.3 mg/g for ^60^Co, and the desorption percent was 95.4%. To form a new nanosorbent (NMn_3_O_4_-OA), manganese oxide nanoparticles were fabricated via combustion synthesis and later functionalization with oleyl amine. The maximum capacities reported in [19], reached 100 mg/g for Co^II^. In another study [20], the nanobentonite clay has been activated with H_2_SO_4_ to produce nanosorbent (A-NBent) with a maximal removal capacity of 125.27 mg/g for ^60^Co.

In the case of ^137^Cs dissolved in surface water reservoir, an illite adsorbent was evaluated by Park et al. [21]. Other scientists analyzed bentonite upon thermal treatment performed at various temperatures, i.e., 150, 300, and 600 °C, considering their effect on the sorption of ^137^Cs [22]. It came out that the heated forms had reduced selectivity for ^137^Cs compared to the original bentonite, while B-150 and B-300 exhibited the best sorbent properties toward ^137^Cs solved in salt media. Magnetic potassium–zinc hexacyanoferrate (II) was investigated by Puzyrnaya et al. [23] to determine its feasibility for the selective sorption of ^137^Cs particles from water. Singh et al. [24] reported that sodium aluminosilicate has been synthesized by the solution route for use as a sorbent for ^137^Cs radionuclides. Voronina et al. [25] studied the uptake of ^137^Cs and ^90^Sr radionuclides out of seawater under batch conditions. They used ferrocyanides based on hydrated titanium and zirconium dioxides (T-35, NPF-HTD), natural aluminosilicates, zirconium phosphate, and modified hydrated titanium dioxide, as well as manganese dioxide based on hydrated titanium dioxide. Nano-sized stannic silicomolybdate was synthesized and tested for the adsorption of ^137^Cs, ^90^Sr, too, as described in [26]. Sadeghi et al. [27] reported the successful synthesis of MnO_2_ NPs-AgX zeolite composite via the impregnation method intended for the uptake of radioactive ^90^Sr from drinking water. The As-obtained novel adsorbent demonstrated high capacity and potential in treatment during 8 h with the yield of 100%. Crystalline silicotitanates (CST) and onosodium titanates (MST) are among the most widely studied materials due to their decent strontium uptake characteristics at neutral and basic conditions, and in [28], it was demonstrated that a novel anionic-layered CP material [(CH3)2NH2][ZrCH2(PO3)2F] (SZ-4) was able to remove Sr^2+^ ions effectively over a very wide pH range, starting from highly acidic environments of pH1 and pH2.

Iron oxides, namely, goethite FeO(OH), maghemite γ-Fe_2_O_3_, hematite α-Fe_2_O_3_, and magnetite Fe_3_O_4_, can be considered promising materials for the uptake of heavy metal ions from aqueous solutions. Among their merits are low costs, availability, good sorption properties, possibility of modification, and lack of toxicity, which was confirmed by numerous studies, e.g., [29,30,31,32,33]. In particular, iron oxides are capable of removing copper, europium, and cerium from aqueous solutions in the range of pH from 5 to 9, reaching a yield of 95–97%. Odnovolova et al. [34] proved that the sorption capacity of α-Fe_2_O_3_ toward europium, cerium, and copper at pH 5 was 21.3, 9.2, and 15.7 mg/g, respectively, while that of Fe_3_O_4_ is 19.7, 7.5, and 11.6 mg/g, respectively. Similarly, it was reported that the sorption capacity of α-Fe_2_O_3_ and Fe_3_O_4_ toward cobalt was close to 18 mg/g [32].

Given the abovementioned needs of new sorbents and proven potential of the iron oxides, it was found reasonable to perform tests on their uptake of radioactive isotopes. In case of radionuclides, a sorption process will take place in the aqueous solution with very small concentration. Thus, the objective of the presented work was to assess the removal effectiveness of hematite α-Fe_2_O_3_ and magnetite Fe_3_O_4_ toward ^60^Co, ^90^Sr, and ^137^Cs.

## 2. Materials and Methods

### 2.1. Synthesis of Powders α-Fe_2_O_3_ and Fe_3_O_4_

The synthesis route of iron oxide micro- and nanopowders affects the final morphology of the particles [35]. The hematite powder α-Fe_2_O_3_ was obtained with the method described in detail in [36]. Namely, to the volume of 100 mL of FeCl_3_ 0.1 M solution was added aqueous solution of ammonia (25% solution 266.8 g/L), reaching pH 10 or 11, and the obtained mixture was agitated with the magnetic stirrer during 15–20 min. The precipitate was filtered, then washed several times using distillated water, and finally calcinated at 450 °C during 4 h. Using the Brunauer–Emmett–Teller (BET) model, it was found that the obtained hematite powder α-Fe_2_O_3_ had a specific surface area (SSA) of 150 m^2^/g.

Similarly, the magnetite Fe_3_O_4_ particles were precipitated from aqueous solution with the method described in [36]. To the Erlenmeyer flask of 500 mL, 100 mL of FeCl_3_ 0.2 M solution and 50 mL of FeSO_4_ 0.2 M solution were added, so that the proportion *c*(Fe^3+^):*c*(Fe^2+^) was 2:1. The mixture was heated up to 80 °C with constant stirring, and then, an aqueous solution of ammonia was added with uninterrupted stirring, reaching pH 10 or 11. The obtained solution was mixed for further 30 min. After that, the precipitate was filtered, washed with distillated water until the ammonia smell disappeared, and then dried up on the air at room temperature during 48 h. The obtained magnetite powder Fe_3_O_4_ had a specific surface area (SSA) of 140 m^2^/g.

### 2.2. Sorption Tests

The sorption capacity of α-Fe_2_O_3_ and Fe_3_O_4_ toward radionuclides ^137^Cs and ^90^Sr was investigated with the limited volume method in the pH interval between 2 and 9 at a temperature of 293 K. In the experiments, model aqueous solutions of radionuclides with no carriers were used, as follows: ^60^Co (5.2 · 10^7^ Bq/dm^3^), ^137^Cs (3.2 · 10^7^ Bq/dm^3^), and ^90^Sr (1.8 · 10^7^ Bq/dm^3^). These solutions were prepared on the basis of distillated water. Sorbent weights of 0.05 g were added to 10 mL of the solution containing radionuclides at known preset pH value. Based on the previous experience, a solid to liquid proportion of 1:200 was then mixed with ultrasonic, but the sorption experiments were performed without stirring or ultrasonic influence.

The time of equilibrium was derived from the sorption kinetics. Kinetic diagrams were built with the method of subsequent samples selection in the time interval from 0 up to τ_∞_ and the measurement of the radioactivity of dry residue from an aliquot of the solution. The τ_∞_ denotes the time of thermodynamic equilibrium in the solution–sorbent system. When two subsequent samples had constant radioactivity, it was seen as the proof of thermodynamic equilibrium in the system.

The radioactivity of the dry residue of the solution aliquot before and after the sorption equilibrium of ^90^Sr was measured with a beta-spectrometer SEB-01-150 produced by PRE Atom Komplex Prylad (Kyiv, Ukraine), while that of ^137^Cs and ^60^Co was measured with an NRR 610 automatic alpha beta counter made by Tesla company (Prague, Czech Republic). The relative error of each measurement did not exceed 2%.

Quantitative characteristics of the interaction between sorbents and radionuclides was determined using sorption percentage coefficient *K_s_*, %. It was calculated as follows:

(1) $$K_{s}=\frac{I_{0}-I_{p}}{I_{0}}\cdot 100\%,$$

where *I*_0_ and *I_p_* denote initial and equilibrium radioactivity of the solution [pps], respectively.

### 2.3. Measurement Apparatus

X-ray powder analysis was performed using Siemens D500 X-ray Diffractometer (XRD), with Cu sources and graphite-diffracted beam monochrometer. Full profile X-ray diagrams were converted in the angle range 10° < 2θ < 90° with a step of 0.02 during 10 s at each point. The infrared spectra were obtained in tablets of potassium bromide with a Spectrum One FT-IR Spectrometer produced by PerkinElmer (Waltham, MA, USA). The sample was thoroughly ground with potassium bromide in a sample/KBr proportion of 1:100, after they were tableted under load 10 t/cm^2^ during 1–2 min.

The morphology of the obtained powder surface was analyzed with a scanning electron microscope (SEM) JSM-6390LV made by Jeol Ltd. (Akishima, Tokyo, Japan) at accelerating voltage 10–20 kV. The samples were placed on the graphite bars of dimensions 5 mm and height 5 mm in form of alcohol suspension, which was later dried in air.

The SSA value of the synthesized powder was obtained with the method of gas thermal desorption with chromatographic detection, using gas mixture 10% of argon in helium. As a reference, alumina with SSA 4.2 and 52 m^2^/g were used.

## 3. Results and Discussion

### 3.1. Characteristics of the Sorbents

**Figure 1:**
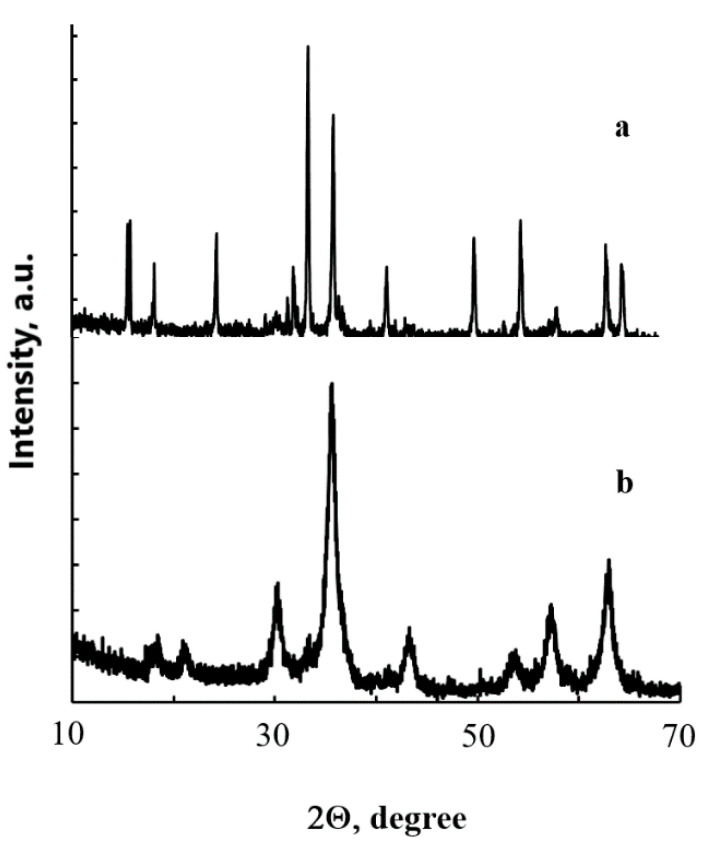
XRD of the synthesized powders α-Fe_2_O_3_ (**a**) and Fe_3_O_4_ (**b**).

Figure 1 presents an XRD measurement of the synthesized iron oxides. In the diagrams, the reflections can be seen that correspond with the respective phases of hematite α-Fe_2_O_3_ (curve a) and magnetite Fe_3_O_4_ (curve b).

**Figure 2:**
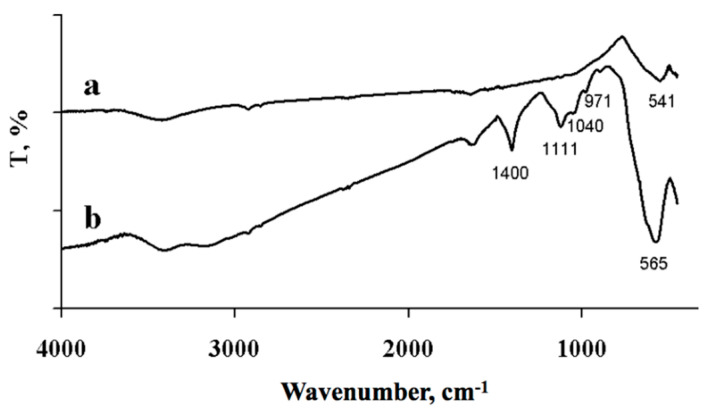
IR spectra of the synthesized powders α-Fe_2_O_3_ (**a**) and Fe_3_O_4_ (**b**).

**Figure 3:**
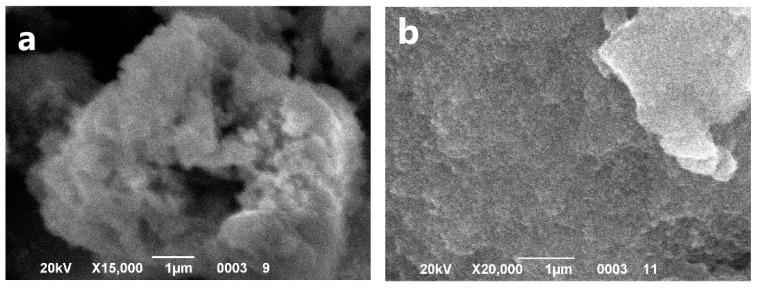
SEM images of the synthesized particles α-Fe_2_O_3_ (**a**) and Fe_3_O_4_ (**b**).

Figure 2 presents the IR spectra of the synthesized iron oxides. In the spectra, intense absorption bands are seen in the range between 500 and 1000 cm^−1^, with the peaks at 541 cm^−1^ (curve *a*) and 565 cm^−1^ (curve *b*). According to several reports [34,37], these peaks are attributed to the vibration of Fe–O bond in the iron oxides α-Fe_2_O_3_ and Fe_3_O_4_, respectively. Moreover, in the IR spectrum of the Fe_3_O_4_ powder represented by curve *b*, absorption bands with maxima at 1400, 1111, 1040, and 971 cm^−1^ are registered, which can be attributed to the presence of SO_4_^2−^. In turn, the absorption band with a maximum of 1400 cm^−1^ can be attributed to the CO_3_^2−^ ion vibrations, as it was in other studies, such as [38,39]. The presence of sulfate ions is caused by the application of iron sulfate as a precursor in the synthesis, while carbonate ions appeared as a consequence of the alkaline solution carbonization process during particles precipitation. SEM images of the obtained particles are presented in Figure 3.

The powder α-Fe_2_O_3_ seen in Figure 3a formed large, porous, shapeless agglomerates with dimensions of ca. 30 μm, which were composed out of small spherical particles below 80 nm. The powder Fe_3_O_4_ appears also in form of porous agglomerates shown in Figure 3b, but their dimensions are much smaller, below 8 μm. It consists of small spherical particles below 80 nm, too.

Synthesized iron oxides were used for further sorption characteristics assessment.

### 3.2. Effect of Sorption Time

**Figure 4:**
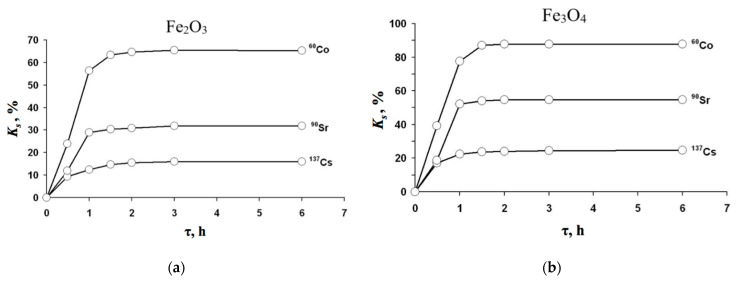
Kinetic sorption diagrams of radionuclides ^60^Co, ^90^Sr, and ^137^Cs at pH 8: (**a**) with particles Fe_2_O_3_; (**b**) with Fe_3_O_4_.

Kinetic investigations were performed in order to determine the optimal time for radionuclides removal. In Figure 4, there are diagrams of the sorption coefficient dependent on the uptake time. From the experimental data, it can be concluded that the sorption coefficient in the time domain behaved similarly for both α-Fe_2_O_3_ and Fe_3_O_4_, and it did not depend on the removed radionuclide ^60^Co, ^90^Sr, and ^137^Cs.

The kinetic diagram may be divided on probation in three areas. The first area of 1 h can be considered as a rapid increase of the *K_s_*, the second one between 1 and 2 h corresponds with its slow increase, and the third one above 3 h is in a stationary area, since it reveals practically no increase of *K_s_*. The latter testifies that the equilibrium is reached.

It should be noted that the maximal sorbent coefficient was reached for ^60^Co, while the next was for ^90^Sr, and the minimal one was for ^137^Cs. At the same time, Fe_3_O_4_ particles performed much higher sorption coefficients than hematite particles. In particular, the sorption ability *K_s_* for Fe_3_O_4_ toward ^60^Co reached 88%, while for α-Fe_2_O_3_ particles, it did not exceed 65%. Similarly, *K_s_* for Fe_3_O_4_ toward ^90^Sr reached 54%, while for α-Fe_2_O_3_ particles, it did not exceed 30%.

From the obtained results, it can be derived that the optimal time for the uptake in the above-mentioned conditions was ca. 1.5 h.

### 3.3. Effect of pH

**Figure 5:**
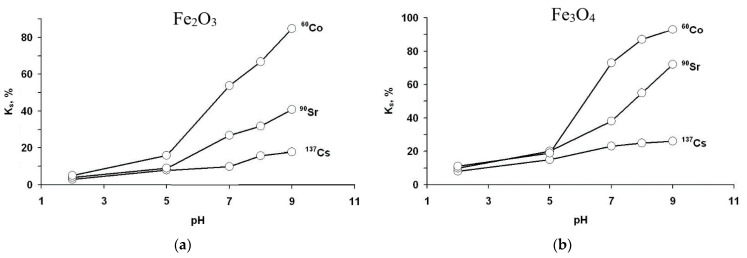
Sorption coefficients *K_s_* of ^60^Co, ^90^Sr, and ^137^Cs dependent on solution pH at temperature 293 K: (**a**) with particles Fe_2_O_3_; (**b**) with Fe_3_O_4_.

Sorption effectiveness is dependent on the solution pH to a high degree. Figure 5 demonstrates the effect of pH on the sorption coefficient *K_s_* during 1.5 h of the experiment. Independent of the nature of iron oxide, the sorption coefficient always gradually increases with the increase of solution pH. In the range of pH 2–5, sorption effectiveness is rather small, and its dependence on pH is weak, since *K_s_* increased from 5–10% to 10–15%. A further pH increase up to 9 led to a substantial improvement of the sorption effectiveness. As in the case of kinetic investigations, the *K_s_* coefficient is the highest for ^60^Co, then for ^90^Sr, and the lowest is for ^137^Cs. The maximal sorption degree was obtained for pH 9, and respective values of the *K_s_* coefficient for ^60^Co, ^90^Sr, and ^137^Cs with hematite particles were *K_s_*_Co_ = 85%, *K_s_*_Sr_ = 41%, and *K_s_*_Cs_ = 18%, while with Fe_3_O_4_ particles, they were *K_s_*_Co_ = 93%, *K_s_*_Sr_ = 73%, and *K_s_*_Cs_ = 26%. It is noteworthy that Fe_3_O_4_ particles performed much higher values of sorption coefficient compared to α-Fe_2_O_3_ particles.

Low sorption effectiveness at pH below 5 may be caused by two factors. First, dependent on the pH, the surface of iron oxides may be loaded positively or negatively as a result of the following reactions:

surface FeOH + H^+^ ↔ surface FeOH^2+^, (2)

surface FeOH + OH^−^ ↔ surface FeO^–^ + H_2_O. (3)

In the acid environments at low pH, iron oxide is loaded positively, which prevents cations uptake concurrent with H^+^ ions. Second, when the pH is below 5, the process of iron oxides dissolution may take place, inhibiting sorption of the radionuclides on the surface.

Increased sorption coefficient at higher pH values is attributed to the possibility of hydrocomplex formation by the metal cations. The relation between cation hydratation energy and sorption effectiveness can be traced, because the hydration energy of Sr^2+^ ions is −∆H^0^_hydr_ = 1412.46 kJ/mol, and that of Co^2+^ ions is −∆H^0^_hydr_ = 1780.68 kJ/mol, which is much higher than that of Cs^+^ ions −∆H^0^_hydr_ = 251.93 kJ/mol reported in [36]. Hence, the higher the hydration energy, the higher the uptake effectiveness.

Moreover, high effectiveness of the tested sorbent toward ^60^Co can be attributed to the predisposition of the Co^2+^ ions to form complex bounds with OH groups of weak coordination bonds. According to [40], the hydration shell Co^2+^ has weak deformation, which determines its stability. As a result, it can be expected that apart from ions exchange, there is a formation of the complex bonds of Co^2+^ ions with weak coordination bonds with OH groups of the sorbent.

It is noteworthy that ^90^Sr uptake is close to the 80% value recently reported by [41] with a composite sorbents containing iron oxide in a polymer matrix after hydrothermal treatment at a temperature of 175 °C. However, in our study, the methodology is much less energy and time consuming.

### 3.4. Drinking Water Treatment

Drinking water components cannot constitute a threat to human health [42]. Elimination of the radionuclides from the natural environment and water is very important due to their harmful effects on living organisms [43]. To make assessment of the radionuclides uptake feasibility in real work conditions, the experiments were performed with the drinking water, pH 6.8. From the measurement results, the uptake coefficient *K_s_*, % toward each radionuclide was calculated. The obtained values are collected in Table 1.

It is noteworthy that the overall trend in drinking water compared to model aqueous solutions is some decrease of the uptake efficiency. This phenomenon can be attributed to the fact that the radionuclides are present in solutions in sub-micron amounts. As a result, the removal takes place concurrently with the ion exchange with a dominant mechanism of secondary potential-forming sorption. Moreover, the presence of extrinsic ions in the drinking water has a further negative impact on the uptake process.

Table 2 presents the chemical composition of the drinking water before and after its treatment with sorbents. It can be seen that the calcium, magnesium, and hydrogen carbonate ions concentration is reduced due to adsorption on oxides.

The hydration energy of the ions Ca^2+^ is 1577 J/mol, while for Mg^2+^, it is 1908 J/mol [40]. Thus, calcium ions interfere with the process of cobalt and strontium ions removal.

## 4. Conclusions

The results of the uptake effectiveness of iron oxides α-Fe_2_O_3_ and Fe_3_O_4_ toward radionuclides ^60^Co, ^90^Sr, and ^137^Cs dissolved in water are very promising. It was found that the sorption efficiency increased with higher pH values. At any pH, the sorption activity of the tested radionuclides fitted to the row as follows: ^60^Co > ^90^Sr > ^137^Cs. It was assumed that the dominant sorption mechanism of these elements on the iron oxides surface was ion exchange.

When applied for drinking water treatment, the tested sorbents had an insufficient decrease of the uptake effectiveness. It was attributed to the concurrent sorption of magnesium and calcium cations.

The results of investigations proved the feasibility of the iron oxides α-Fe_2_O_3_ and Fe_3_O_4_ as sorbents in water treatment plants. They may become an alternative because of their low costs, availability, modification flexibility, and lack of toxicity. From the perspective of green and sustainable manufacturing, the iron oxides appeared to be advantageous materials.

## Figures and Tables

**Table 1:** Sorption effectiveness of investigated sorbents applied in drinking water.

Sorbent	Sorption Coefficients toward Respective Radionuclide *K_s_*, %		
	^137^Cs	^90^Sr	^60^Co
α-Fe_2_O_3_	8.7%	19.8%	51.4%
Fe_3_O_4_	18.7%	34.8%	71.6%

**Table 2:** Chemical composition of the drinking water before and after its treatment with respective sorbents α-Fe_2_O_3_ and Fe_3_O_4_.

Indicators	Content before and after Treatment, mg/dm^3^		
	Before	After α-Fe_2_O_3_	After Fe_3_O_4_
Sodium (Na^+^)	93.0	92.7	92.5
Potassium (K^+^)	13.0	12.3	12.0
Magnesium (Mg^2+^)	24.3	14.2	13.9
Calcium (Ca^2+^)	86.2	27.6	27.1
Sulfates (SO_4_^2−^)	107.5	104.9	104.5
Hydrogen carbonates (HCO_3_^−^)	293.0	157.1	147.0
Chlorides (Cl^−^)	160.0	156.0	156.0

## Data Availability

Data available on request due to privacy restrictions.
